# Multimodal Data Fusion for Precise Lettuce Phenotype Estimation Using Deep Learning Algorithms

**DOI:** 10.3390/plants13223217

**Published:** 2024-11-15

**Authors:** Lixin Hou, Yuxia Zhu, Mengke Wang, Ning Wei, Jiachi Dong, Yaodong Tao, Jing Zhou, Jian Zhang

**Affiliations:** 1 College of Information and Technology, Jilin Agricultural University, Changchun 130118, China; lixinh@jlau.edu.cn (L.H.); 20221152@mails.jlau.edu.cn (Y.Z.); 20241615@mails.jlau.edu.cn (M.W.); 20231296@mails.jlau.edu.cn (N.W.); 2212030329@mails.jlau.edu.cn (J.D.); 2 School of Computer Science and Technology, Beijing Jiaotong University, Beijing 100044, China; ydtao@bjtu.edu.cn; 3 Faculty of Agronomy, Jilin Agricultural University, Changchun 130118, China; 4 Department of Biology, University of British Columbia, Okanagan, Kelowna, BC V5K1K5, Canada

**Keywords:** deep learning, phenotype, lettuce, RGB-D

## Abstract

Effective lettuce cultivation requires precise monitoring of growth characteristics, quality assessment, and optimal harvest timing. In a recent study, a deep learning model based on multimodal data fusion was developed to estimate lettuce phenotypic traits accurately. A dual-modal network combining RGB and depth images was designed using an open lettuce dataset. The network incorporated both a feature correction module and a feature fusion module, significantly enhancing the performance in object detection, segmentation, and trait estimation. The model demonstrated high accuracy in estimating key traits, including fresh weight (fw), dry weight (dw), plant height (h), canopy diameter (d), and leaf area (la), achieving an R^2^ of 0.9732 for fresh weight. Robustness and accuracy were further validated through 5-fold cross-validation, offering a promising approach for future crop phenotyping.

## 1. Introduction

Lettuce, *Lactuca sativa* L., is a commercially crucial leafy vegetable rich in vitamins, carotenoids, dietary fiber, and other trace elements [1]. Global lettuce consumption has increased rapidly in recent years due to its high nutritional and medicinal value [2,3]. Although lettuce has a rapid growth rate [4] and multiple harvesting times, it is sensitive to its growth environment. For example, it has poor adaptability to saline–alkali soil [5], and different light environments can affect its growth morphology and nutrient content [6,7]. It is vital to carefully monitor the crops during critical growth stages to maintain consistent supply and quality.

Plant phenotypic analysis is an interdisciplinary research field. Plant phenotypic information reflects various traits of the whole life cycle, such as growth form, development process, physiological response, etc. These traits result from interactions between plant genotypes and environmental conditions [8,9]. Linking phenotypic traits to genotypes can help select high-yield, stress-resistant varieties, thereby improving agricultural productivity to meet the demands of growing populations and climate change [10]. One of the significant challenges in crop breeding is the imperfect phenotypic detection technology [11]. Traditional phenotypic monitoring relies on visual observation and manual measurement, which is time-consuming and error-prone, and it needs to be more accurate in evaluating trait diversity among different varieties. Therefore, automated phenotyping technologies are essential for more efficient and accurate plant trait detection.

Recent advancements in computer vision, algorithms, and sensors have significantly progressed plant phenotypic analysis. Many imaging techniques can now capture complex traits associated with growth, yield, and adaptation to biotic or abiotic stresses, such as disease, insect infestation, water stress, and nutrient deficiencies. These techniques include digital imaging spectroscopy, chlorophyll fluorescence, thermal infrared, RGB, and 3D imaging [12,13]. Spectral images can be utilized to analyze the physiological characteristics of lettuce, such as the leaf’s overall physiological condition, water content, pigment, and structural composition information related to biomass [14]. Eshkabilov et al. [15] employed hyperspectral data and artificial neural network (ANN) to predict the fresh weight, chlorophyll, sugar, vitamins, and nutrients of lettuce, achieving an R index ranging from 0.85 to 0.99. Yu et al. [16] used hyperspectral data and time series phenotype as input, combined with RNN and CNN models, to detect SSC, pH, nitrate, calcium, and water stress levels of lettuce. Based on hyperspectral images, Ye et al. [17] estimated the total chlorophyll of greenhouse lettuce, and the average R^2^ and RMSE were 0.746 and 2.018. Hyperspectrum contains more information than multispectrum, but the data processing is more complex, and the equipment is expensive.

The canopy area was estimated by chlorophyll fluorescence imaging to predict fresh weight, and heavier lettuce or anthocyanins impacted the results [18,19]. Thermal infrared imaging can obtain the temperature of the plant or leaf, generally as supplementary data. Concepcion et al. [20] combined thermal imaging and RGB images to estimate lettuce’s full moisture content and equivalent water thickness. The R^2^ scores reached 0.9233 and 0.8155, respectively.

RGB imaging is the most commonly used method for crop phenotype studies due to its low cost, ease of use, and simple data processing [21,22,23,24,25]. Yu et al. [21] collected multi-view images of lettuce under water and nitrogen stress and used ConvLSTM to predict the images of lettuce. RMSE, SSIM, and PSNR results were 0.0180, 0.9951, and 35.4641, respectively. The average error of the phenotypic geometric index based on prediction images was less than 0.55%. Zhang et al. [22] employed a CNN with RGB images to estimate three lettuce types’ fresh weight, dry weight, and leaf area. They achieved R^2^ values of 0.8938, 0.8910, and 0.9156, respectively, with NRMSE values of 26.00%, 22.07%, and 19.94%. Three-dimensional imaging could provide more information than RGB imaging by capturing an object’s three-dimensional coordinate information and generating its stereoscopic image [26,27,28]. Lou et al. [26] used a ToF camera to capture point cloud data from a top-down perspective, and the lettuce point cloud was reconstructed using geometric methods. The results showed that the completed point cloud had a high linear correlation with actual plant height (R^2^ = 0.961), leaf area (R^2^ = 0.964), and fresh weight (R^2^ = 0.911).2

As can be seen from the above studies, phenotypic analysis based on a single mode has accumulated many research results. Still, the information provided by a single sensor needs to be improved. The multimodal information has a certain degree of complementarity and consistency, which can compensate for each other’s shortcomings. Using multimodal data to improve model performance has become popular in lettuce phenotype research.

The fusion methods for different modal information can be divided into three categories: data layer fusion, feature layer fusion, and decision layer fusion. The data layer fusion method treats multimodal data as indistinguishable multichannel data. It can use the inherent complementarity between modes to supplement the incomplete information in the input stage [29]. Taha et al. [30] combined spectral vegetation indices and color vegetation indices to estimate the chlorophyll content of hydroponic lettuce. The AutoML model outperformed the traditional model with an R^2^ of 0.98.

The feature layer fusion method integrates multimodal images into parallel branches, extracts independent features of different scales, and performs feature fusion. Wu et al. [31] proposed a hybrid model based on dual-transformer and convolutional neural networks to detect lettuce phenotypic parameters using RGB-D image data. The average R^2^ of phenotypic traits was 92.47.

The decision-level fusion method is the fusion of the detection results of the previous stage [32,33,34,35]. In the study by Lin et al. [32], the U-Net model was used to segment lettuce, extract leaf boundary and geometric features, and estimate fresh weight through a multi-branch regression network fusion of RGB images, depth images, and geometric features. The experimental results showed that the multimodal fusion model significantly improved the accuracy of lettuce fresh weight estimation in different growth periods. The RMSE of the whole growth period was 25.3 g, and the R^2^ was 0.938.

Using feature layer fusion methods with multimodal data has been relatively rare in the lettuce phenotyping field. A new multimodal fusion method based on the feature layer was proposed to address this gap, which mainly performed feature extraction and fusion for RGB and depth image data. The main contributions are as follows: (1) A feature correction module is proposed, which filters and corrects each other’s feature noise information in channel and spatial dimensions, based on the principle that the information and noise of different modes are usually complementary. (2) A feature fusion module based on SE attention is proposed to integrate the features of the two models into a unified feature map. (3) The phenotypic trait header uses a residual structure, replacing linear interpolation in the feature pyramid network (FPN) with transposed convolution. The experimental results showed that the model improved lettuce’s object detection and segmentation performance and performed well in estimating phenotypic traits.

## 2. Materials and Methods

### 2.1. Dataset

**Figure 1:**
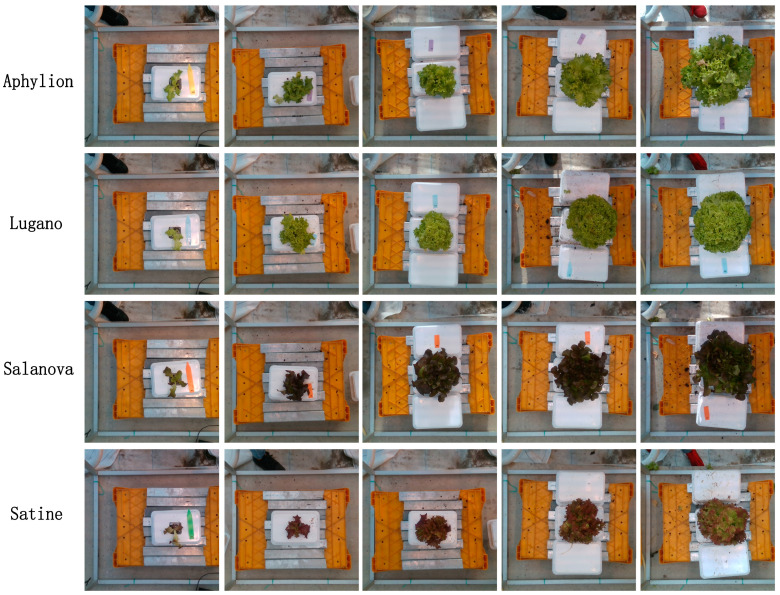
Images of the four types of lettuce.

The data used in the experiment was from the open dataset of Tencent and Wageningen University’s third Autonomous Greenhouse Challenge [36]. It included images and measurements of four lettuce varieties (Lugano, Salanova, Aphylion, and Satine) grown under controlled greenhouse conditions. The RealSense D415 depth sensor (Intel, Santa Clara, CA, USA), suspended 0.9 m above the crops, captured RGB and depth images with a resolution of 1920 × 1080. A total of 96, 102, 92, and 98 image pairs were taken over six weekly intervals for each variety. The four varieties of lettuce are shown in Figure 1.

Fresh weight, dry weight, plant height, canopy diameter, and leaf area were obtained by destructive measurement. The fresh weight was obtained by measuring the weight of the lettuce harvested from the first leaf attachment point, and the dry weight was measured after the fresh weight was obtained and dried in the oven for 3d. Leaf area was calculated by projecting the surface area onto the plane after separating the leaf from the stem without considering the increase in leaf area due to leaf curvature. The diameter of the lettuce projected onto the plane was measured, and the height was measured from the point where the first leaf was connected to the highest point of the plant in units of “g/plant”, “g/plant”, “cm”, “cm”, and “cm^2^”.

#### Data Preprocessing

**Figure 2:**
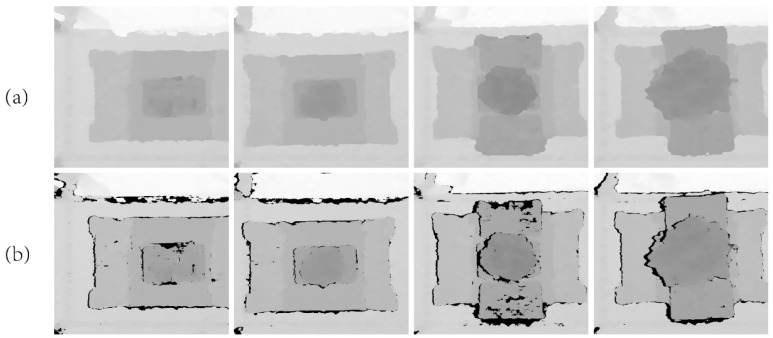
Depth images. (**a**) The completed image; (**b**) the image before completion.

Depth images taken directly from the camera are often missing, noisy, or sparse. Using these incomplete data for deep learning training can lead to reduced model performance and instability. Deep image completion significantly enhances data quality, coherence, and consistency, providing higher-quality input for deep learning models. The depth completion method proposed by Ku et al. [37] was used to repair the dataset’s depth images. The deep completion algorithm first inverts pixel values by $D_{inverted}=100.0-D_{input}$, then uses the 5 × 5 rhombic kernel to expand them. Secondly, they are processed with small hole closure, small hole filling, extension to the top of the frame, large hole filling, median, and Gaussian blur. Finally, $D_{output}=100.0-D_{inverted}$ is used to revert to the original depth encoding. This algorithm relies solely on traditional image processing techniques, does not require training, and is robust against overfitting. Figure 2 compares the completed images with the original images.

**Figure 3:**
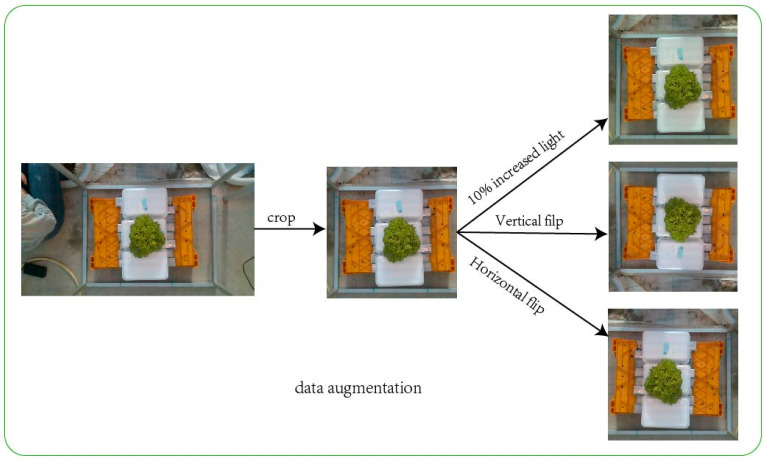
The preprocessing process of the dataset.

All images were cropped from the center to 1024 × 1024 pixels and then resized to 800 × 800 pixels. The VIA annotation tool was used to manually label the dataset images, where each image pair contained a lettuce target, categorized into four types: Lugano, Salanova, Aphylion, and Satine. Data augmentation was applied to improve the network’s learning and generalization ability, including horizontal and vertical flips and a 10% increase in brightness. The dataset augmentation process is shown in Figure 3. After data augmentation, the dataset is 1548 images. The K-fold cross-validation method was used, dividing the data into five subsets. For each training session, one fold was designated as the test set, while the remaining four were used for training, with 10% of the training set reserved for validation.

### 2.2. Method

**Figure 4:**
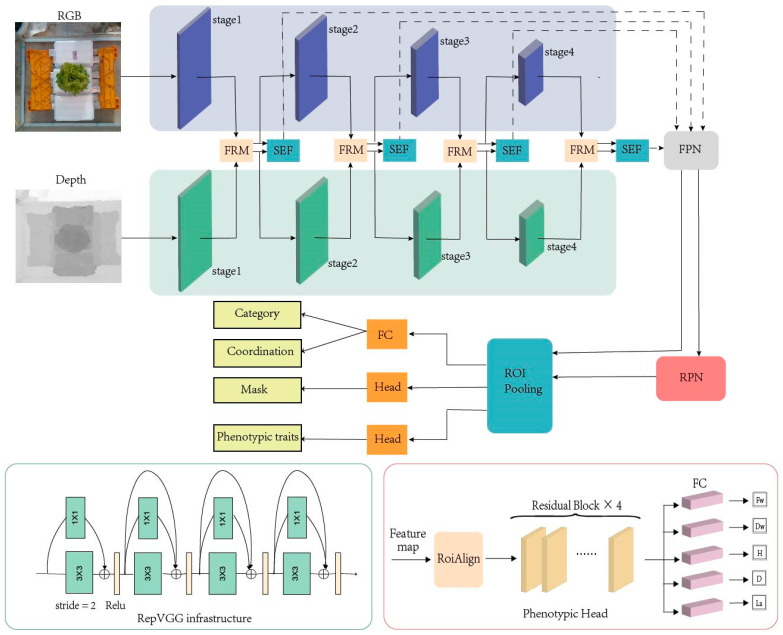
The overall structure of the model.

The overall architecture of the proposed model framework is shown in Figure 4, which is based on MaskRCNN. The backbone segment consists of two RepVGG networks, one accepting RGB image input and the other receiving depth image input. The RepVGG creation is inspired by ResNet, a multi-branch structure that uses identity, 3 × 3, and 1 × 1 branches during training. The RepVGG infrastructure is shown in the lower-left corner of Figure 4. The features of different levels of each backbone network are input to the feature correction module, and the two corrected features are sent to the next stage and input to the feature fusion module simultaneously. Feature fusion module fuses the two modal features of the same stage into feature maps. Each layer of fusion features will be input into the improved feature network pyramid, and the output features will be processed like the classic MaskRCNN process to obtain the final result.

#### 2.2.1. FRM

**Figure 5:**
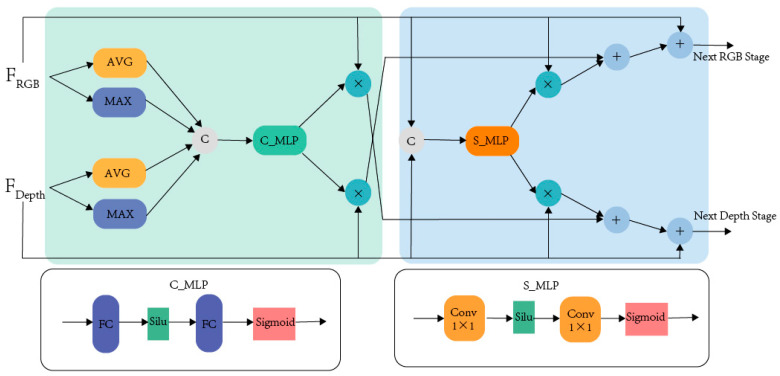
Structure of FRM module.

RGB features contain a lot of color and texture information, while depth features focus on spatial position information. Although the information concerns of the two modes are different, the information of the different modes is usually complementary, and the noise is the same [38,39]. Therefore, this feature filters and calibrates the noise information between the features. CM-FRM was proposed by Zhang et al. [40] in 2023. Figure 5 shows the structure of the FRM. The module is divided into two parts, dealing with input parallel flow features in spatial and channel dimensions. In this study, we adjusted the original basis by replacing the activation function from Relu to Silu. We set the dimensionality reduction ratio of the first fully connected layer/convolution layer in the channel and spatial correction module to one-quarter.

First, the details of channel feature correction are introduced. We connect bimodal features along the channel and apply global maximum pooling and global average pooling to the connected features. After concatenating the above results, C_MLP is applied. The C_MLP contains two fully connected layers and activation functions. The first fully connected layer reduces the number of channels to 1/4 dimension, and the next fully connected layer increases the number of channels to 1/2 dimension. Equation (1) is expressed as

(1) $$W_{RGB}^{C},W_{D}^{C}=f_{split}(\sigma {(FC}_{2}(Silu\left({FC}_{1}\left(W_{avg}©W_{max}\right)\right)))$$

$W_{avg}$ and $W_{max}$ represent the features after concatenating input features and applying adaptive average pooling and adaptive maximum pooling, respectively. © means concatenate. σ denotes the Sigmoid function. Then, the Sigmoid function is used, and the result is split to $W_{RGB}^{C}$ and $W_{D}^{C}$. The dual-modal features are concatenated along the channel in the spatial feature correction and fed into S_MLP. S_MLP has the same structure as C_MLP except for replacing the fully connected layer with the convolution layer. The features of the S_MLP output are further divided into two weight graphs. Equation (2) is expressed as follows:

(2) $$W_{RGB}^{S},W_{D}^{S}=f_{split}(\sigma ({Conv}_{1\times 1}\left(Silu\left({Conv}_{1\times 1}\left(F_{RGB}^{in}©F_{D}^{in}\right)\right)\right)))$$

$F_{RGB}^{in}$ and $F_{D}^{in}$ represent the RGB and depth characteristics of the input. The channel weights and spatial weights obtained through the above process are multiplied by the corresponding elements of the input modal features and then added to the input bimodal features. Equations (3) and (4) are expressed as follows:

(3) $$F_{RGB}^{out}=F_{RGB}^{in}+{0.5F}_{D}^{in}*W_{D}^{C}+{0.5F}_{D}^{in}*W_{D}^{S}$$

(4) $$F_{D}^{out}=F_{D}^{in}+{0.5F}_{RGB}^{in}*W_{RGB}^{C}+{0.5F}_{RGB}^{in}*W_{RGB}^{S}$$

#### 2.2.2. SEF

**Figure 6:**
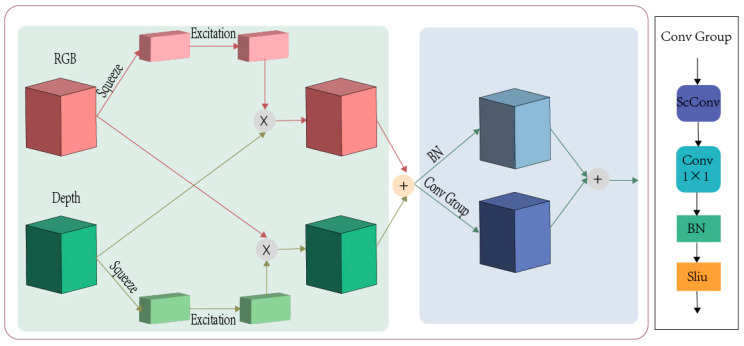
Structure of SEF module.

We designed a feature fusion module to facilitate information exchange and integrate the features of the two modes into a unified feature map. This approach allows for better integration of information from different modalities, enhancing the model’s performance. Since the feature fusion module applies SE attention, it is called Squeeze-and-Excitation Fusion (SEF). Figure 6 shows the structure of the SEF. SE attention was proposed in 2019 and has inspired many subsequent attention mechanisms. The operation of SE is simple: applying global average pooling to the features, then using the two-modal attention weight obtained by the fully connected layer, and cross-multiplying it with the two-modal input features. Equation (5) is expressed as

(5) $$W_{mid}=F_{RGB}^{in}*W_{D}^{SE}+F_{D}^{in}*W_{RGB}^{SE}$$

In the next stage, we used operations similar to the residual structure to integrate the dual-modal features further. One branch consists of SCConv, 1 × 1 convolution, BN, and Silu activation functions, and the other branch has only one BN. SCConv was proposed in 2023 [41]. SCConv is designed to reduce redundant computing and consists of a spatial reconstruction unit (SRU) and a channel reconstruction unit (CRU). Using SCConv can reduce redundant features and better fuse features to improve performance. Equation (6) is expressed as follows:

(6) $$F_{merge}^{out}=Silu(BN({Conv}_{1\times 1}\left(ScConv\left(W_{mid}\right)\right)))+BN(W_{mid})$$

#### 2.2.3. Other Improvement Points

Feature pyramid network (FPN) uses a top-down architecture combined with horizontal connections to build high-level semantic feature maps at all scales, significantly improving multi-scale object detection performance. The network replaces linear interpolation with transposed convolution to further boost performance, allowing for more efficient recovery of high-resolution feature maps. This method can retain more spatial information and improve the model’s accuracy in object detection and segmentation tasks.

Depth images are grayscale images in which each pixel value represents the distance from the camera to the object, and they contain less information than RGB images. To extract features from depth images better, convolution kernels of different scales were used. Parallel 1 × 1 and 3 × 3 convolution was added to the header of the backbone network, and the resulting features were superimposed.

It was found that the phenotypic branch head used eight convolutional layers to extract RGB features well, but the results were poor after the fusion of RGB images and depth images. To improve the prediction effect of phenotypic traits, 8-layer convolution was replaced with the residual structure. The structure of the Phenotypic Head is shown in the lower-right corner of Figure 4. The number of parameters and GFLOPs remained unchanged, but the experimental result was improved.

#### 2.2.4. Training Strategy and Experimental Environment

The model training process began by training the overall model and then all layers of the backbone network while freezing the other network layers. Next, only all branch subnetworks were trained, including detection, segmentation, and phenotype branches. Finally, other parts of the network were frozen. Only the first two stages of the backbone network, which contain corresponding FRM and SEF modules, were trained.

The initial learning rate of this study was 0.0012. Each new training session reduced the learning rate to one-tenth of the original. The batch size was 6, the total number of training rounds was 140, and the optimizer was Adam. This study used the Ubuntu 22.04.3 operating system with a kernel of 6.5.0-35-generic. The computer configurations included an NVIDIA GeForce RTX 4070 Ti graphics card (12 GB VRAM), 32 GB RAM, and an Intel^®^ CoreTM i7-13700KF processor. The model was written in Python 3.8 and pytorch1.10. The details are listed in Table 1. All experiments were trained and evaluated using the same hardware setup to compare their performance fairly.

## 3. Results

### 3.1. Evaluation Index

In this study, COCO evaluation indexes were used for detection and segmentation. The COCO index is a mainstream evaluation criterion for object detection and segmentation, including AP (Average Accuracy) and AR (Average Recall). It uses ten different IoU thresholds (0.5 to 0.95, separated by 0.05) to assess how closely the detection box or segmentation mask matches the actual annotation. The primary metric for COCO is AP0.5:0.95, which is the AP average across all categories and all IoU thresholds. In addition, we also introduce F1, which is an essential indicator for evaluating the performance of binary classification models, especially in the case of class imbalance. It is the harmonic average of Precision and Recall, considering the performance of these two metrics. Equation (7) for calculating F1 is

(7) $$F1=2\times \frac{Precision\times Recall}{Precision+Recall}$$

Precision refers to the proportion of all samples predicted by the model to be positive that are positive. Recall is the percentage of all positive samples the model correctly predicts will be positive.

In the regression prediction of lettuce phenotypic traits, R^2^, MAPE, and NRMSE indexes were used. R^2^ is the evaluation index of regression analysis, representing the proportion of all the variation in the dependent variable that the independent variable can explain through the regression relationship. The range is [0, 1]. The closer R^2^ is to 1, the better the model fit; the closer R^2^ is to 0, the worse the fit. Equation (8) for calculating R^2^ is

(8) $$R^{2}=1-\frac{\sum_{i}{\left(y_{i}-y_{i}^{\prime }\right)}^{2}}{\sum_{i}{\left(y_{i}-\bar{y}\right)}^{2}}$$

In the formula, $\bar{y}$ is the average of the actual value, $y_{i}^{\prime }$ is the i-th model predicted value, and $y_{i}$ is the i-th true value.

MAPE (Mean Absolute Percentage Error) represents the relative difference between predicted and actual values. It provides a percentage that indicates the accuracy of a model. The range of MAPE is from 0 to infinity. A MAPE of 0% indicates a perfect model, while higher percentages indicate a less effective model. Equation (9) for calculating MAPE is as follows:

(9) $$MAPE=\frac{1}{n}\sum_{i=1}^{n}\left|\frac{y_{i}-y_{i}^{\prime }}{y_{i}}\right|\times 100\%$$

In the formula, $y_{i}^{\prime }$ is the *i*-th model predicted value, and $y_{i}$ is the *i*-th true value.

NRMSE (Normalized Root Mean Square Error) is derived by normalizing the square root of the Mean Squared Error (MSE). The value of NRMSE ranges from 0 to 1. MSE represents the average of the squares of the differences between predicted and actual values. Unlike MAPE, NRMSE emphasizes the impact of more significant errors. Equation (10) for NRMSE is as follows:

(10) $$NRMSE=\frac{\sqrt{\frac{1}{n}\sum_{i=1}^{n}{\left(y_{i}^{\prime }-y_{i}\right)}^{2}}}{\max\left(y\right)-min(y)}$$

In the formula, $y_{i}^{\prime }$ is the *i*-th model predicted value, $y_{i}$ is the *i*-th true value, and $\max\left(y\right)/min(y)$ indicates the largest/smallest value out of the true values.

### 3.2. Model Performance

**Figure 7:**
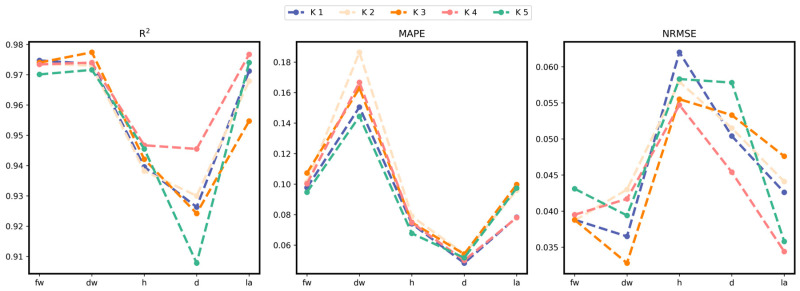
Line charts of the R^2^, MAPE, and NRMSE metrics for the five phenotypes in the 5-fold cross-validation experiment.

The test set results are shown in Figure 7 and Table 2, Table 3 and Table 4. The three subgraphs in Figure 7 correspond to the evaluation scores of R^2^, MAPE, and NRMSE indicators in fw, dw, h, d, and la growth traits, and each subgraph contains the results of the K-fold cross-validation method. Bold fonts in all tables represent the best results.

When R^2^ is 0–1, the larger the R^2^ value, the better the estimation effect. The smaller the MAPE and NRMSE values, the better the network performance. Our proposed method performs well in various indicators, among which the predicted R^2^ of fw, dw, h, d, and la are 0.9732, 0.9739, 0.9424, 0.9268, and 0.9689, respectively, and MAPE is 0.1003, 0.1622, 0.074, 0.0516 and 0.0864. NRMSE is 0.0398, 0.0387, 0.0577, 0.0517 and 0.0409. In the standard deviation results, d’s R^2^ and dw and la’s MAPE standard deviation are greater than 0.01, and the rest are less than 0.01.

As can be seen from Figure 1, the R^2^ results of the 5-fold cross-validation experiment of fw and dw are the closest and highest, while the R^2^ results of d are the lowest and most dispersed. However, among the MAPE error values, dw is the largest but most dispersed, and d is the smallest. The maximum error value of NRMSE is h, and the minimum error value is dw. While the R^2^ of fw and dw is the highest and closest, the MAPE of dw (0.1622) is significantly higher than that of fw (0.1003), suggesting that while the model explained the overall change in dw well, there were large relative errors in some predictions.

MAPE is sensitive to negligible values and may perform poorly when dealing with negative or near-zero scenes. The early growth value of dw is small, which may be one of the reasons for the high MAPE value. The R^2^ of h is relatively high, but the prediction error of NRMSE is greatest in 5 phenotypic traits. The R^2^ of d shows some fluctuation, indicating that the prediction fit degree fluctuates, but MAPE and NRMSE both show minor and stable prediction errors. The R^2^ value of la is 0.9689, the MAPE is 0.0864, and the NRMSE is 0.0409, all showing high Precision and low error. The model is the most robust in la prediction, with high accuracy and consistency.

The model’s object detection and segmentation results on the lettuce dataset are shown in Table 5. The average AP50:95, AP50, and AP75 of the object detection results of the 5-fold cross-validation experiment are 0.8881, 0.9979, and 0.9945, respectively, and the average AP50:95, AP50:95, and AP75 of the segmentation results are 0.9041, 0.9979, 0.9969, respectively. As you can see, the fourth fold is the best result in the phenotypic trait prediction section. However, the second fold of object detection and segmentation has the best effect.

**Figure 8:**
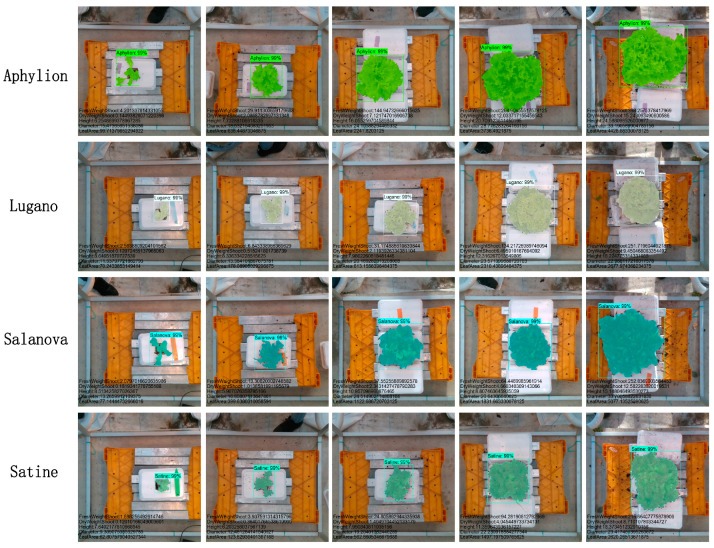
Visualization results of the model.

The inference results of the four varieties of lettuce model are shown in Figure 8. It can be seen from the figure that no matter the type of lettuce, the early segmentation effect is better, and different lettuce shows different forms as it grows. Both Lugano and Satine show leaf clumping and folding at the later stage of growth, but Satine has more prominent small leaf folds than Lugano, and the effect is relatively poor at the edges. The leaves of Salanova are more spread than other varieties, and careful observation shows that the effect is indeed the worst in the later stage. In Aphylion and Salanova, it can be observed that less of the edge is not covered, which means that the edge covers more of the non-leaf position, decreasing segmentation results, as evidenced by the indicators in the Section 4.2.

## 4. Discussion

### 4.1. Ablation Experiment

In this section, we performed ablation experiments on all the proposed modules. The results are shown in Table 6 and Table 7. The bold font indicates the best results. The first line indicates that the model data input is only RGB data, and the specific experimental details can be seen [42]. The second row of data represents a simple addition of the corresponding elements only for RGB and depth data. After introducing the depth image, the object detection and segmentation indexes are significantly improved, indicating that depth information is crucial to enhancing the performance of object detection and segmentation. However, the phenotypic trait index is lower than that of only RGB images, which means that although RGB and depth images complement each other, they also introduce many redundant data. After adding the FRM and SEF modules, the highest AP50:95 score for object detection and segmentation is obtained. The R^2^ scores of phenotypic traits are improved, but d and h are still lower than those of only RGB input. The values of MAPE and NRMSE generally decline compared to the simple addition of RGB and depth data. Although the depth of information enhances the model’s performance in some aspects, there may be information redundancy in the evaluation of phenotypic traits, which affects the overall effect of the model.

According to the data in the last four rows, the most noticeable improvement in the score of phenotypic traits occurs when replacing the convolutional layer of the phenotypic trait head with a residual structure. Compared with only RGB input, the R^2^ scores of fw and dw are more than 0.97, the R^2^ of d and h increase by nearly 0.01, and the values of MAPE and NRMSE also generally decrease. These results show that the residual structure can capture critical information more effectively and reduce information loss during feature extraction, thus enhancing the model’s overall performance. Although the introduction of the improved FPN and replacement with the residual structure (i.e., line 7) obtained the highest AP50 and AP75, our final model achieves the best overall result in the score of the phenotypic trait index. Moreover, the overall effect of target detection and segmentation can be ranked second. In summary, the results of this ablation experiment not only validate the importance of depth information in target detection and segmentation tasks but reveal the critical impact of feature extraction structure on the evaluation of phenotypic traits. These findings provide a valuable basis for optimizing subsequent models, and future work can further explore how to more effectively combine data from different modes to achieve more comprehensive and accurate phenotypic analysis.

### 4.2. Category Analysis

**Figure 9:**
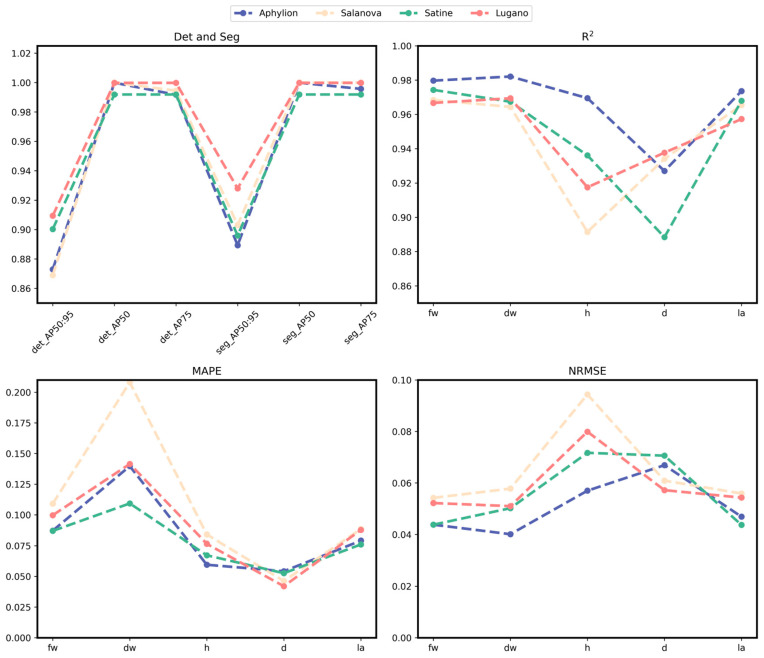
Line charts of object detection, segmentation, and phenotypic trait scores for the four lettuce varieties.

To analyze the model’s performance on different types of lettuce in the dataset, we detected and segmented four varieties and calculated their phenotypic traits. The index lines of the four kinds of lettuce are presented in Figure 9. The detection’s AP50:95 scores for the four lettuce categories are generally lower than those for segmentation, with the AP50 scores being consistent. Notably, the AP75 detection scores for Aphylion and Salanova are lower than their segmentation scores, while the scores for the other two varieties remain consistent. This score is consistent with what we see with our naked eyes. Although Lugano, Aphylion, and Satine differ in color and shape, they exhibit more curled and folded leaves, resulting in an overall clumped growth pattern. In contrast, Salanova and Satine share similar colors but have significant differences in their leaf structures; Salanova features flat leaves that are relatively elongated. Consequently, despite Salanova being the most prevalent variety in the dataset, its evaluation index score is the lowest among the four categories.

In terms of phenotypic parameter prediction, it can be seen from the figure that there are differences in the results obtained by different varieties. The R^2^ scores of dry and fresh weight are higher than 0.96, and Aphylion shows the best performance: 0.9797 and 0.9821, respectively. Salanova’s R^2^ score in h and Satine’s R^2^ score in d are only 0.90 or less. MAPE and NRMSE show the same trend as the K-fold cross-validation results in five traits. Salanova performs poorly in all other traits except for the top two evaluation indexes of the d trait. On the contrary, Satine has the top two traits except d. Overall, Salanova is the worst performer of the four categories.

### 4.3. Comparison with Other Modules

In order to thoroughly verify that our proposed module has excellent effects on the detection, segmentation, and prediction of phenotypic traits of lettuce, we conducted a comparative experiment with SAGate, which was proposed by Chen et al. [38], CM-FRM and FFM proposed by Zhang et al. [40], CRM proposed by Ji et al. [43], and RGB-D Fusion proposed by Seichter et al. [44]. Table 8 and Table 9 show the results of the average value of the five-fold cross-validation experiment for each module. The best result of each column is marked in bold. As seen from Table 8, although our model is not optimal in terms of speed, AP50:95 for both detection and segmentation is optimal. Table 9 shows the results of phenotypic trait indicators. The suboptimal phenotypes rank second and have little difference from the optimal results. Our proposed module achieves significantly better results than other modules in most indicators, and only the MAPE index of dw is slightly lower than other methods but still maintains a relatively close performance. Although the running time is not dominant, our indicators are generally optimal in all aspects. Subsequent research can improve the lightweight module.

## 5. Conclusions

In this study, we proposed a method for detecting lettuce objects, segmenting images, and estimating phenotypic traits by fusing multimodal features from RGB and depth images. We employed a dual-flow convolutional neural network to extract multi-scale features from both modes. We used feature rectification and fusion modules for efficient information interaction and integration. The AP50:95 scores of the model in object detection and segmentation of lettuce were 0.8881 and 0.9041, respectively. Furthermore, it performed exceptionally well in estimating fresh weight, dry weight, plant height, canopy diameter, and leaf area, with predicted R^2^ values for fresh and dry weight reaching 0.9732 and 0.9739, respectively. The experimental results show that the fusion of multimodal data can make up for the limitation of single-modal data and improve the prediction accuracy and robustness of the model. Future research could focus on optimizing feature fusion strategies and expanding the dataset to include various environmental conditions and phenotypic analyses of other crops. At the same time, we are developing a Raspberry Pi-based lettuce phenotype estimation system to provide an accurate growth condition monitoring solution for lettuce cultivation in greenhouses. This study provides a new technical approach and practical foundation for phenotypic monitoring in crop breeding and agricultural production.

## Figures and Tables

**Table 1:** Training configuration parameters and hardware configuration of the experiment.

Training Configuration Parameters		Hardware Configuration	
Epoch	140	CPU	Intel^®^ CoreTM i7-13700KF
Original_lr	0.0012	GPU	NVIDIA GeForce RTX 4070 Ti
Batch size	6	Operating System	Ubuntu 22.04.3
Optimizer	Adam	RAM	32 GB

**Table 2:** R^2^ results for the five phenotypes in the 5-fold cross-validation experiment.

NE	fw	dw	h	d	la
k1	**0.9747**	0.9736	0.9396	0.9263	0.9712
k2	0.9736	0.9730	0.9382	0.9300	0.9679
k3	0.9740	**0.9774**	0.9421	0.9242	0.9547
k4	0.9734	0.9740	**0.9467**	**0.9455**	**0.9767**
k5	0.9701	0.9716	0.9455	0.9078	0.9740
average	0.9732	0.9739	0.9424	0.9268	0.9689
std_dev	0.0018	0.0021	0.0037	0.0135	0.0086

NE refers to the number of experiments. The std_dev means standard deviation.

**Table 3:** MAPE results for the five phenotypes in the 5-fold cross-validation experiment.

NE	fw	dw	h	d	la
k1	0.0977	0.1505	0.074	0.0482	0.0783
k2	0.1014	0.1864	0.0789	0.0542	0.0959
k3	0.1073	0.1629	0.0749	0.0541	0.0997
k4	0.1003	0.1667	0.0747	**0.05**	**0.078**
k5	**0.0947**	**0.1444**	**0.0677**	0.0517	0.0802
average	0.1003	0.1622	0.074	0.0516	0.0864
std_dev	0.0047	0.0163	0.0040	0.0026	0.0105

NE refers to the number of experiments. The std_dev means standard deviation.

**Table 4:** NRMSE results for the five phenotypes in the 5-fold cross-validation experiment.

NE	fw	dw	h	d	la
k1	0.0388	0.0365	0.062	0.0504	0.0426
k2	**0.0387**	0.043	0.0579	0.0515	0.0441
k3	0.0388	**0.0328**	0.0555	0.0533	0.0476
k4	0.0395	0.0417	**0.0547**	**0.0454**	**0.0344**
k5	0.0431	0.0394	0.0583	0.0578	0.0358
average	0.0398	0.0387	0.0577	0.0517	0.0409
std_dev	0.0019	0.0041	0.0029	0.0045	0.0056

NE refers to the number of experiments. The std_dev means standard deviation.

**Table 5:** Object detection and segmentation results for the five phenotypes in the 5-fold cross-validation experiment.

NE	DEL					SEG				
	AP50:95	AP50	AP75	F1	AR	AP50:95	AP50	AP75	F1	AR
k1	0.8829	0.9899	0.9846	0.9501	0.913	0.9002	0.9899	0.9846	0.9578	0.928
k2	**0.9013**	0.9999	0.993	**0.9617**	**0.9263**	**0.906**	0.9999	0.9999	0.9645	0.9316
k3	0.8875	**1**	**1**	0.9564	0.9165	0.9028	**1**	**1**	0.9625	0.9278
k4	0.8971	0.9998	0.9998	0.9606	0.9244	0.9059	0.9998	0.9998	0.9637	0.9302
k5	0.8716	**1**	0.995	0.9494	0.9037	0.9058	**1**	**1**	**0.9653**	**0.933**
average	0.8881	0.9979	0.9945	0.9556	0.9168	0.9041	0.9979	0.9969	0.9628	0.9301
std_dev	0.0118	0.0044	0.0063	0.0057	0.0091	0.0026	0.0045	0.0069	0.0030	0.0023

NE refers to the number of experiments. AR stands for Average Recall. The std_dev means standard deviation.

**Table 6:** Ablation experiment results for object detection and segmentation, and R^2^ scores for phenotypic traits.

RGB	Depth	FRM	SEF	D_Pre	I_FPN	R_Head	DEL			SEG			R^2^				
							AP50:95	AP50	AP75	AP50:95	AP50	AP75	fw	dw	h	d	la
√							0.8684	0.9964	0.9854	0.8804	0.9964	0.9933	0.96	0.9596	0.9329	0.9136	0.9592
√	√						0.8804	0.9955	0.9908	0.8973	0.9955	0.9955	0.9597	0.9593	0.9056	0.8731	0.9566
√	√	√					0.8881	0.9969	0.995	0.9041	0.9969	0.9969	0.9628	0.9609	0.9093	0.8791	0.9605
√	√	√	√				**0.889**	0.9968	0.9958	**0.9055**	0.9968	0.9968	0.9655	0.9636	0.9076	0.8828	0.9596
√	√	√	√	√	√		0.8838	0.9958	0.9944	0.9041	0.9958	0.9958	0.963	0.9648	0.9109	0.884	0.9579
√	√	√	√	√		√	0.8836	0.9948	0.9927	0.9002	0.9948	0.9917	0.9726	0.9727	0.9413	0.9229	0.9649
√	√	√	√		√	√	0.8872	**0.9983**	**0.9978**	0.9043	**0.9983**	**0.9983**	0.9727	0.9732	**0.9436**	0.9229	0.9639
√	√	√	√	√	√	√	0.8881	0.9979	0.9945	0.9041	0.9979	0.9969	**0.9732**	**0.9739**	0.9424	**0.9268**	**0.9689**

D_Pre, I_FPN, and R_Head represent the multi-scale convolution kernels added in front of the depth backbone network, the improved FPN, and the phenotypic trait head using a residual structure, respectively. The symbol ‘√’ indicates that the model introduces this operation.

**Table 7:** Ablation experiment results for MAPE and NRMSE scores for phenotypic traits.

RGB	Depth	FRM	SEF	D_Pre	I_FPN	R_Head	MAPE					NRMSE				
							fw	dw	h	d	la	fw	dw	h	d	la
√							0.1072	0.1522	0.0757	0.0548	0.0899	0.0508	0.0518	0.0649	0.0634	0.0548
√	√						0.1227	0.1721	0.1001	0.0704	0.1081	0.0485	0.0483	0.0737	0.0688	0.0486
√	√	√					0.1152	0.1631	0.0958	0.0675	0.0995	0.0463	0.047	0.0717	0.0664	0.046
√	√	√	√				0.1181	0.1739	0.098	0.0667	0.1029	0.0449	0.0458	0.0729	0.0659	0.047
√	√	√	√	√	√		0.11731	0.1725	0.0981	0.0677	0.1049	0.047	0.0452	0.0719	0.0659	0.0478
√	√	√	√	√		√	0.1104	0.1744	**0.0715**	0.0520	0.0935	0.04	0.0396	0.0581	0.0535	0.0436
√	√	√	√		√	√	**0.0971**	**0.1551**	0.0724	0.0524	0.0871	0.04	0.0393	**0.0569**	0.0534	0.0443
√	√	√	√	√	√	√	0.1003	0.1622	0.074	**0.0516**	**0.0864**	**0.0398**	**0.0387**	0.0577	**0.0517**	**0.0409**

D_Pre, I_FPN, and R_Head represent the multi-scale convolution kernels added in front of the depth backbone network, the improved FPN, and the phenotypic trait head using a residual structure, respectively. The symbol ‘√’ indicates that the model introduces this operation.

**Table 8:** Results of object detection and segmentation compared with other modules.

Module	DEL			SEG			Params(MB)	Time(s)
	AP50:95	AP50	AP75	AP50:95	AP50	AP75		
ours	**0.8881**	**0.9979**	0.9945	**0.9041**	**0.9979**	**0.9969**	229.1	0.0281
Chen et al. [38]	0.881	0.9973	**0.9964**	0.89936	0.9973	0.9954	167.1	**0.0195**
Zhang et al. [40]	0.8779	0.9928	0.9886	0.89496	0.9928	0.9912	454.9	0.0301
Ji et al. [43]	0.8583	0.9947	0.9869	0.895	0.9947	0.9929	191.5	0.0207
Seichter et al. [44]	0.8765	0.9975	0.9908	0.8961	0.9975	0.9956	**166.2**	0.0207

**Table 9:** Results of R^2^, MAPE, and NRMSE scores for phenotypic traits compared with other modules.

Module	R^2^					MAPE					NRMSE				
	fw	dw	h	d	la	fw	dw	h	d	la	fw	dw	h	d	la
ours	**0.9732**	**0.9739**	**0.9424**	**0.9268**	**0.9689**	**0.1003**	0.1622	**0.074**	**0.0516**	**0.0864**	**0.0398**	**0.0387**	**0.0577**	**0.0517**	**0.0409**
Chen et al. [38]	0.967	0.9644	0.9297	0.9181	0.9632	0.1059	0.1635	0.0796	0.0531	0.091	0.0435	0.0448	0.0633	0.0543	0.0443
Zhang et al. [40]	0.9703	0.9680	0.9316	0.9130	0.9610	0.1077	0.1612	0.0806	0.0580	0.0941	0.0419	0.0429	0.0624	0.0565	0.0455
Ji et al. [43]	0.9647	0.9673	0.9268	0.9169	0.9616	0.11	0.1716	0.0829	0.0545	0.0933	0.045	0.0432	0.0645	0.0544	0.0455
Seichter et al. [44]	0.9701	0.9718	0.9365	0.9153	0.9641	0.1032	**0.1547**	0.0744	0.0531	0.0898	0.0414	0.0396	0.0601	0.0550	0.0438

## Data Availability

This study used the Third Autonomous Greenhouse Challenge: Online Challenge Lettuce Images dataset publicly available at 4TU.ResearchData [36].

## References

[1] Kim M.J., Moon Y., Tou J.C., Mou B., Waterland N.L. (2016). Nutritional value, bioactive compounds and health benefits of lettuce (*Lactuca sativa* L.). J. Food Compos. Anal..

[2] Eshkabilov S., Lee A., Sun X., Lee C.W., Simsek H. (2021). Hyperspectral imaging techniques for rapid detection of nutrient content of hydroponically grown lettuce cultivars. Comput. Electron. Agric..

[3] Noumedem J.A.K., Djeussi D.E., Hritcu L., Mihasan M., Kuete V., Kuete V. (2017). Chapter 20—*Lactuca sativa*. Medicinal Spices and Vegetables from Africa.

[4] Grahn C.M., Benedict C., Thornton T., Miles C. (2015). Production of Baby-leaf Salad Greens in the Spring and Fall Seasons of Northwest Washington. HortScience.

[5] Adhikari N.D., Simko I., Mou B. (2019). Phenomic and physiological analysis of salinity effects on lettuce. Sensors.

[6] Matysiak B., Ropelewska E., Wrzodak A., Kowalski A., Kaniszewski S. (2022). Yield and Quality of Romaine Lettuce at Different Daily Light Integral in an Indoor Controlled Environment. Agronomy.

[7] Lin K.-H., Huang M.-Y., Huang W.-D., Hsu M.-H., Yang Z.-W., Yang C.-M. (2013). The effects of red, blue, and white light-emitting diodes on the growth, development, and edible quality of hydroponically grown lettuce (*Lactuca sativa* L. var. *capitata*). Sci. Hortic..

[8] Hickey L.T., Hafeez A.N., Robinson H., Jackson S.A., Leal-Bertioli S.C.M., Tester M., Gao C., Godwin I.D., Hayes B.J., Wulff B.B.H. (2019). Breeding crops to feed 10 billion. Nat. Biotechnol..

[9] Abebe A.M., Kim Y., Kim J., Kim S.L., Baek J. (2023). Image-Based High-Throughput Phenotyping in Horticultural Crops. Plants.

[10] Yang G., Liu J., Zhao C., Li Z., Huang Y., Yu H., Xu B., Yang X., Zhu D., Zhang X. (2017). Unmanned Aerial Vehicle Remote Sensing for Field-Based Crop Phenotyping: Current Status and Perspectives. Front. Plant Sci..

[11] Großkinsky D.K., Svensgaard J., Christensen S., Roitsch T. (2015). Plant phenomics and the need for physiological phenotyping across scales to narrow the genotype-to-phenotype knowledge gap. J. Exp. Bot..

[12] Shakoor N., Lee S., Mockler T.C. (2017). High throughput phenotyping to accelerate crop breeding and monitoring of diseases in the field. Curr. Opin. Plant Biol..

[13] Zhao C., Zhang Y., Du J., Guo X., Wen W., Gu S., Wang J., Fan J. (2019). Crop Phenomics: Current Status and Perspectives. Front. Plant Sci..

[14] Zhang C., Marzougui A., Sankaran S. (2020). High-resolution satellite imagery applications in crop phenotyping: An overview. Comput. Electron. Agric..

[15] Eshkabilov S., Simko I.J.A. (2024). Assessing Contents of Sugars, Vitamins, and Nutrients in Baby Leaf Lettuce from Hyperspectral Data with Machine Learning Models. Agriculture.

[16] Yu S., Fan J., Lu X., Wen W., Shao S., Liang D., Yang X., Guo X., Zhao C. (2023). Deep learning models based on hyperspectral data and time-series phenotypes for predicting quality attributes in lettuces under water stress. Comput. Electron. Agric..

[17] Ye Z., Tan X., Dai M., Chen X., Zhong Y., Zhang Y., Ruan Y., Kong D. (2024). A hyperspectral deep learning attention model for predicting lettuce chlorophyll content. Plant Methods.

[18] Simko I., Hayes R.J., Furbank R.T. (2016). Non-destructive Phenotyping of Lettuce Plants in Early Stages of Development with Optical Sensors. Front. Plant Sci..

[19] Kim C., van Iersel M.W. (2022). Morphological and Physiological Screening to Predict Lettuce Biomass Production in Controlled Environment Agriculture. Remote Sens..

[20] Concepcion R., Lauguico S., Almero V.J., Dadios E., Bandala A., Sybingco E. Lettuce Leaf Water Stress Estimation Based on Thermo-Visible Signatures Using Recurrent Neural Network Optimized by Evolutionary Strategy. Proceedings of the 2020 IEEE 8th R10 Humanitarian Technology Conference (R10-HTC).

[21] Yu H., Dong M., Zhao R., Zhang L., Sui Y. (2024). Research on precise phenotype identification and growth prediction of lettuce based on deep learning. Environ. Res..

[22] Zhang L., Xu Z., Xu D., Ma J., Chen Y., Fu Z. (2020). Growth monitoring of greenhouse lettuce based on a convolutional neural network. Hortic. Res..

[23] Islam S., Reza M.N., Chowdhury M., Ahmed S., Lee K.-H., Ali M., Cho Y.J., Noh D.H., Chung S.-O. (2024). Detection and segmentation of lettuce seedlings from seedling-growing tray imagery using an improved mask R-CNN method. Smart Agric. Technol..

[24] Tan J., Hou J., Xu W., Zheng H., Gu S., Zhou Y., Qi L., Ma R. (2023). PosNet: Estimating lettuce fresh weight in plant factory based on oblique image. Comput. Electron. Agric..

[25] Ruan A., Xu M., Ban S., Wei S., Tian M., Yang H., Hu A., Hu D., Li L. (2024). LettuceNet: A Novel Deep Learning Approach for Efficient Lettuce Localization and Counting. Agriculture.

[26] Lou M., Lu J., Wang L., Jiang H., Zhou M. (2022). Growth parameter acquisition and geometric point cloud completion of lettuce. Front. Plant Sci..

[27] Li J., Wang Y., Zheng L., Zhang M., Wang M. (2023). Towards end-to-end deep RNN based networks to precisely regress of the lettuce plant height by single perspective sparse 3D point cloud. Expert Syst. Appl..

[28] Mortensen A.K., Bender A., Whelan B., Barbour M.M., Sukkarieh S., Karstoft H., Gislum R. (2018). Segmentation of lettuce in coloured 3D point clouds for fresh weight estimation. Comput. Electron. Agric..

[29] Shuai L., Chen Z., Li Z., Li H., Zhang B., Wang Y., Mu J. (2023). Real-time dense small object detection algorithm based on multi-modal tea shoots. Front. Plant Sci..

[30] Taha M.F., Mao H., Wang Y., ElManawy A.I., Elmasry G., Wu L., Memon M.S., Niu Z., Huang T., Qiu Z. (2024). High-Throughput Analysis of Leaf Chlorophyll Content in Aquaponically Grown Lettuce Using Hyperspectral Reflectance and RGB Images. Plants.

[31] Wu Z., Liu X., Xue Y., Wen J., Peng W. HDTC: Hybrid Model of Dual-Transformer and Convolutional Neural Network from RGB-D for Detection of Lettuce Growth Traits. Proceedings of the 2023 IEEE International Conference on Image Processing (ICIP).

[32] Lin Z., Fu R., Ren G., Zhong R., Ying Y., Lin T. (2022). Automatic monitoring of lettuce fresh weight by multi-modal fusion based deep learning. Front. Plant Sci..

[33] Zhang Q., Zhang X., Wu Y., Li X. (2022). TMSCNet: A three-stage multi-branch self-correcting trait estimation network for RGB and depth images of lettuce. Front. Plant Sci..

[34] Gang M.-S., Kim H.-J., Kim D.-W. (2022). Estimation of Greenhouse Lettuce Growth Indices Based on a Two-Stage CNN Using RGB-D Images. Sensors.

[35] Ojo M.O., Zahid A., Masabni J.G. (2024). Estimating hydroponic lettuce phenotypic parameters for efficient resource allocation. Comput. Electron. Agric..

[36] Hemming S., de Zwart H.F., Elings A., Bijlaard M., van Marrewijk B., Petropoulou A. 3rd Autonomous Greenhouse Challenge: Online Challenge Lettuce Images. https://data.4tu.nl/articles/_/15023088/1.

[37] Ku J., Harakeh A., Waslander S.L. In defense of classical image processing: Fast depth completion on the CPU. Proceedings of the 2018 15th Conference on Computer and Robot Vision (CRV).

[38] Chen X., Lin K.-Y., Wang J., Wu W., Qian C., Li H., Zeng G. Bi-directional Cross-Modality Feature Propagation with Separation-and-Aggregation Gate for RGB-D Semantic Segmentation. Proceedings of the European Conference on Computer Vision.

[39] Hu X., Yang K., Fei L., Wang K. Acnet: Attention based network to exploit complementary features for RGBD semantic segmentation. Proceedings of the 2019 IEEE International Conference on Image Processing (ICIP).

[40] Zhang J., Liu H., Yang K., Hu X., Liu R., Stiefelhagen R. (2023). CMX: Cross-Modal Fusion for RGB-X Semantic Segmentation with Transformers. IEEE Trans. Intell. Transp. Syst..

[41] Li J., Wen Y., He L. SCConv: Spatial and Channel Reconstruction Convolution for Feature Redundancy. Proceedings of the IEEE/CVF Conference on Computer Vision and Pattern Recognition (CVPR).

[42] Hou L., Zhu Y., Wei N., Liu Z., You J., Zhou J., Zhang J. (2024). Study on Utilizing Mask R-CNN for Phenotypic Estimation of Lettuce’s Growth Status and Optimal Harvest Timing. Agronomy.

[43] Ji W., Li J., Yu S., Zhang M., Piao Y., Yao S., Bi Q., Ma K., Zheng Y., Lu H. Calibrated RGB-D salient object detection. Proceedings of the IEEE/CVF Conference on Computer Vision and Pattern Recognition.

[44] Seichter D., Köhler M., Lewandowski B., Wengefeld T., Gross H.-M. Efficient RGB-D Semantic Segmentation for Indoor Scene Analysis. Proceedings of the 2021 IEEE International Conference on Robotics and Automation (ICRA).

